# Mycoplasma infection as an inhibitory factor for developing DIHS/DRESS

**DOI:** 10.1186/2045-7022-4-S3-P149

**Published:** 2014-07-18

**Authors:** Yoshiko Mizukawa, Ryo Takahashi, Yoko Kano, Tetuo Shiohara

**Affiliations:** 1 Kyorin University School of Medicine, Dermatology, Japan; 2 Kyorin University School of Medicine, Division of Flow Cytometry, Japan

## 

Mycoplasma infection is a well-known etiologic agent of Stevens-Johnson syndrome (SJS)/toxic epidermal necrolysis (TEN). However, the effect of mycoplasma infection on drug-induced hypersensitivity syndrome/ drug rash and eosinophilia with systemic symptoms (DIHS/DRESS) has not been clarified. We report a 25-year-old female suspected of having DIHS/DRESS with a high fever and erythematous eruption affecting the face, neck, and trunk. Four weeks prior to skin rash, the patient had been prescribed carbamazepine for epilepsy. On day 26 of her medication use, the eruption began with erythema on her neck, gradually worsening over the next 3 days. The patient was admitted to our hospital under suspicion of DIHS/DRESS. Her white blood cell count was 3900/L with eosinophilia (13.9%). Liver function tests revealed an increase in serum ALT and AST levels (ALT: 177 IU/L, AST: 124 IU/L), and hypogammaglobulinemia was detected (IgG: 939 mg/dL). The patient had experienced a severe bout of coughing 10 days before admission, and mycoplasma infection was confirmed based on an increase in mycoplasma antibody titers. LTT was positive at an earlier point (7th hospital day) than is typically observed with DIHS/DRESS. Surprisingly, the patient rapidly recovered after only discontinuation of carbamazepine administration. Our previous study demonstrated that regulatory T (Treg) cells with suppressive function were expanded at the acute stage of DIHS/DRESS, while functional defects in Treg cells were detected in patients with SJS/TEN. We therefore investigated whether Treg cells obtained from this patient with an abortive form of DIHS/DRESS were able to retain their suppressive capacity. Our findings showed that Treg cells from our present patient were profoundly defective in their ability to suppress T cell proliferation, demonstrating that mycoplasma infection was indeed able to suppress Treg function. In view of these findings, we suggest that mycoplasma infection be deemed a risk factor for developing SJS/TEN but be accepted as important for inhibiting development of DIHS/DRESS.

